# Sexual behaviour and smoking as determinants of cervical HPV infection and of CIN3 among those infected: a case–control study nested within the Manchester cohort

**DOI:** 10.1054/bjoc.2000.1523

**Published:** 2000-11-22

**Authors:** J M Deacon, C D Evans, R Yule, M Desai, W Binns, C Taylor, J Peto

**Affiliations:** Section of Epidemiology, Institute of Cancer Research, 15 Cotswold Road, Belmont, Sutton, Surrey, SM2 5NG; Department of Cytology, Christie Hospital NHS Trust, Wilmslow Road, Withington, Manchester, M20 4BX, UK

**Keywords:** cervical neoplasia, CIN3, HPV, sexual behaviour, smoking

## Abstract

To distinguish risk factors for acquisition of cervical human papillomavirus (HPV) infection from the determinants of neoplasia among infected individuals we have conducted a three-arm case-control study nested within a large population-based cohort of women (the Manchester cohort) screened for HPV at entry using L1 consensus primer PCR. The study includes 181 HPV-positive controls who did not develop high-grade cervical intraepithelial neoplasia (CIN3) during follow-up, 203 HPV-negative controls, and 199 HPV-positive cases with histologically confirmed CIN3. Detailed information on sexual, reproductive and gynaecological history, oral contraceptive use and smoking was obtained at face-to-face interview. There was a striking division between risk factors for infection and those predictive of disease. Comparing the HPV-positive against the HPV-negative controls, the only risk factors for infection were number of sexual partners (OR for six or more = 3.89; 95% Cl = 1.99–7.62), a relatively recent new sexual relationship (OR for a new partner within the previous 2 years = 4.17; 95% Cl = 2.13–8.33), and a history of previous miscarriage (OR = 2.59; 95% Cl = 1.28–5.21). The determinants of CIN3 among infected women were, in contrast, early age at first intercourse (OR for 16 years old or less = 3.23; 95% Cl = 1.33–7.69), a long time since starting a new sexual relationship (OR for 6 years or more = 4.94; 95% Cl = 2.51–9.71), and cigarette smoking, with strong evidence for a dose– response (OR for current smoking habit 20+ per day = 2.57; 95% Cl = 1.49–4.45). Oral contraceptive use was not significantly associated with either HPV infection or CIN3. © 2000 Cancer Research Campaign http://www.bjcancer.com


*British Journal of Cancer* (2000) **88**(11), 1565–1572
© 2000 Cancer Research Campaign
doi: 10.1054/ bjoc.2000.1523, available online at http://www.idealibrary.com on IDEAL®

http://www.bjcancer.com

### Sexual behaviour and smoking as determinants of cervical HPV infection and of CIN3 among those infected: a case–control study nested within the Manchester cohort

**JM Deacon[1], CD Evans[1], R Yule[2], M Desai[2], W Binns[1], C Taylor[1] and J Peto[1]**

[1]Section of Epidemiology, Institute of Cancer Research, 15 Cotswold Road, Belmont, Sutton, Surrey, SM2 5NG; [2]Department of Cytology, Christie Hospital NHS Trust, Wilmslow Road, Withington, Manchester, M20 4BX, UK

Summary To distinguish risk factors for acquisition of cervical human papillomavirus (HPV) infection from the determinants of neoplasia among infected individuals we have conducted a three-arm case-control study nested within a large population-based cohort of women (the Manchester cohort) screened for HPV at entry using L1 consensus primer PCR. The study includes 181 HPV-positive controls who did not develop high-grade cervical intraepithelial neoplasia (CIN3) during follow-up, 203 HPV-negative controls, and 199 HPV-positive cases with histologically confirmed CIN3. Detailed information on sexual, reproductive and gynaecological history, oral contraceptive use and smoking was obtained at face-to-face interview. There was a striking division between risk factors for infection and those predictive of disease. Comparing the HPV-positive against the HPV-negative controls, the only risk factors for infection were number of sexual partners (OR for six or more = 3.89; 95% CI = 1.99–7.62), a relatively recent new sexual relationship (OR for a new partner within the previous 2 years = 4.17; 95% CI = 2.13–8.33), and a history of previous miscarriage (OR = 2.59; 95% CI = 1.28–5.21). The determinants of CIN3 among infected women were, in contrast, early age at first intercourse (OR for 16 years old or less = 3.23; 95% CI = 1.33–7.69), a long time since starting a new sexual relationship (OR for 6 years or more = 4.94; 95% CI = 2.51–9.71), and cigarette smoking, with strong evidence for a dose–response (OR for current smoking habit 20+ per day = 2.57; 95% CI = 1.49–4.45). Oral contraceptive use was not significantly associated with either HPV infection or CIN3. © 2000 Cancer Research Campaign http://www.bjcancer.com

**Keywords**: cervical neoplasia; CIN3; HPV; sexual behaviour; smoking

Human papillomavirus (HPV) infection has now been accepted as the primary cause of cervical neoplasia (Schiffman, 1992; Bosch et al, 1995; IARC, 1995; Walboomers et al, 1999). Oncogenic genital HPVs, however, are highly prevalent in many populations where CIN3 and cervical cancer remain relatively rare (Bauer et al, 1991, 1993; Hildesheim et al, 1993; Melkert et al, 1993; Wheeler et al, 1993) and natural history studies have shown that most infections are transient and not associated with detectable cytological abnormality (Evander et al, 1995; Hinchliffe et al, 1995; Ho et al, 1998). The reasons for this variable natural history are poorly understood but it has been generally assumed that other causes or co-factors must be important for the development of neoplasia in HPV-infected individuals. In addition to sexual activity, case-control studies have identified a number of other possible factors including high parity, the presence of other chronic genital infections, cigarette smoking and oral contraceptive use (Brinton, 1992). These effects may, however, be due at least partly to the correlation between sexual behaviour and other risk factors, and the failure in most studies to control adequately for confounding by HPV (Layde, 1989; Phillips and Davey Smith, 1994).

In an attempt to resolve these problems, and to distinguish between risk factors for the acquisition of cervical HPV infection and co-factors for the development of CIN3 among those infected, we have conducted a three-arm interview-based case-control study in which the basis for case and control selection was HPV infection as well as disease status. The study was nested in a large population-based cohort undergoing routine cervical screening in the city of Manchester, where cervical cell samples for HPV analysis had been collected from all participants at entry (Peto et al, in preparation). It includes 199 cases of histologically confirmed CIN3 diagnosed after entry to the cohort in women known to be HPV-positive, 181 women infected with HPV but without CIN3 and 203 HPV-negative controls.

*Received 8 June 2000*
*Revised 31 August 2000*
*Accepted 31 August 2000*

*Correspondence to:* JM Deacon

### MATERIALS AND METHODS

### The cohort

The recruitment of the Manchester cohort and the methods of data collection and follow-up are described elsewhere (Peto et al, in preparation). Briefly, between 1987 and 1993, in collaboration with over 100 general practitioners and screening clinics in the Greater Manchester area who used the Christie Hospital cytology service, we collected 78 062 cervical cell samples from 61 570 women attending for routine screening. The cell samples were obtained by elution from spatulas used to take Pap smears. There was no age restriction for participants. All samples were allocated a unique identifying number (ID) and were stored in buffer at –40˚C. Through regular data linkage of the study IDs with the main laboratory database, the screening records of cohort members and the histology results of any cervical biopsies have been updated.

### Case-control study – eligibility criteria

This study was restricted to white women of any age who had been routinely and satisfactorily screened at least once through the Christie laboratory prior to entry to the main cohort, and who had had a smear and concurrent spatula cell sample (termed ‘spatula’) taken on or after 1 July 1988, that were diagnostically adequate both for cytology and for PCR. These samples were the ‘index’ smear and spatula that defined a woman’s age and HPV status. Women were excluded if they had had a hysterectomy, a previous malignancy or previous CIN3, if they suffered from serious psychiatric illness, or if they were known to have moved out of the study area since entry to the cohort. Because of the temporary nature of their residence in the city, students at Manchester’s several universities and colleges of further education were also excluded. The cytological result of the index smear was not a criterion for determining either case or control eligibility.

### Case identification

Cases were all those women in the cohort, eligible by the above criteria, who developed histologically diagnosed CIN3 within 3 years of their index smear.

### Control selection

Two control groups of approximately equal size were identified, the first consisting of women found, on PCR screening of their index spatulas, to be HPV-positive, and the second consisting of HPV-negative women. No control had CIN3 diagnosed at any time during her follow-up but women with CIN1 or 2 were not excluded. Potential controls were identified through stratified random sampling. All the spatulas collected since 1 July 1988 from eligible women in the cohort were stratified by date (1-year periods: 1 July 1988 to 30 June 1989, etc) and age of the woman at the time (5-year age groups: < 20, 20–24, 25–29, etc). Within each age/period stratum spatulas were submitted for HPV testing in random order. Testing continued until the number of HPV-positive spatulas identified roughly equalled the number of cases in each stratum. In instances where a woman had two or more spatulas independently selected for random testing, the earlier was defined as her index. Controls approached for interview were the first HPV-positive and HPV-negative women in their stratum to be identified by the random HPV testing.

### HPV detection and genotyping

HPV DNA in the index spatulas of cases and the random potential controls was assayed at the Institute of Cancer Research using L1 consensus PCR amplification with the MY09/MY11 primer pair as described in detail elsewhere (Bauer et al, 1992; Manos et al, 1989). Concurrent amplification of a 286 bp human β-globin fragment was used as an internal control, its failure to amplify designating a sample inadequate. Consensus HPV-product was detected using a biotinylated generic probe and enhanced chemiluminescence (Amersham International). Positive samples were hybridized with type-specific oligonucleotide probes to determine the virus genotypes present (types 6/11/42 (mixed), 16, 18, 26, 31, 33, 35, 39, 40, 45, 51, 52, 53, 54, 55, 56, 57, 58, 59, 73, ME180, PAP88 (HPV66), PAP155, PAP291 and W13B). Samples positive to the generic probe but negative to all type-specific probes were designated as positive, but untyped.

### Interviews

The ID numbers of potentially eligible cases and controls with adequate HPV results were identified to the Christie Hospital, where eligibility was checked as far as was possible from routine records. A senior clinician (RY) then contacted the general practitioner of each woman to obtain approval for an interviewer to contact the patient and invite her to participate. Between August 1990 and July 1996 interviews were conducted in the women’s homes by one of two trained interviewers using a structured questionnaire. Information collected included demographic data, menstrual, reproductive and gynaecological history, sexual history and information on smoking habits. Sexual, contraceptive and reproductive histories were collected on a calendar with a record for every month of a woman’s life from the age of menarche (or first intercourse, whichever was earlier) up to the date of index smear. Inconsistent data were rechecked with study participants by telephone. The data were then anonymized, coded and entered on computer.

### Statistical analysis

All risk factor analyses relate to exposures prior to the index spatula date. Two separate analyses were performed. The first, analysing risk factors for the acquisition of HPV infection, compared HPV-positive controls with HPV-negative controls. The second, analysing risk factors for the development of CIN3 among those infected with HPV, compared the CIN3 cases with the HPV-positive controls. The age stratification of the study design was maintained in univariate and multivariate conditional logistic regression analyses (Breslow and Day, 1980) using the STATA for Windows® program package. Relative risks were estimated as odds ratios (OR) with approximate 95% confidence intervals. $\chi^2$ values for differences are Mantel–Haenszel corrected. Tests for linear trend of log odds were performed by scoring categories of exposure and fitting them as continuous variables. All *P* values are two-sided.

### RESULTS

315 potential cases and 583 potential controls were identified by the HPV laboratory and determined from the cytology database and GP records to be eligible. Of these, 232 cases (74%) and 384 controls (181 HPV-positive and 203 HPV-negative, total 66%)

**Table 1** Age distributions of interviewed women by case/control status

| Age | HPV+ve CIN3 Cases | | HPV+ve Controls | | HPV–veControls | |
|---|---|---|---|---|---|---|
| | (*n*) | (%) | (*n*) | (%) | (*n*) | (%) |
| < 20 | 4 | (2.0) | 7 | (3.8) | 5 | (2.5) |
| 20–24 | 19 | (9.6) | 19 | (10.5) | 21 | (10.3) |
| 25–29 | 52 | (26.1) | 47 | (26.0) | 49 | (24.1) |
| 30–34 | 49 | (24.6) | 40 | (22.1) | 47 | (23.2) |
| 35–39 | 32 | (16.1) | 29 | (16.0) | 32 | (15.8) |
| 40–44 | 23 | (11.6) | 19 | (10.5) | 21 | (10.3) |
| 45–49 | 7 | (3.5) | 9 | (5.0) | 12 | (5.9) |
| 50+ | 13 | (6.5) | 11 | (6.1) | 16 | (7.9) |
| Total | 199 | | 181 | | 203 | |

© 2000 Cancer Research Campaign

**Table 2** Characteristics of cases and controls: univariate odds ratios[a] for HPV infection among controls, and CIN3 among those infected

| | HPV+ve Controls vs HPV−ve Controls | | | | HPV+ve Cases vs HPV+ve Controls | | | |
|---|---|---|---|---|---|---|---|---|
| | +ve | –ve | OR | 95% CI | Ca | Con | OR | 95%CI |
| Marital status | | | | | | | | |
| Married | 83 | 130 | 1.00 | | 92 | 83 | 1.00 | |
| Cohabiting | 26 | 19 | 2.02[b] | (1.03–3.98) | 35 | 26 | 1.22 | (0.67–2.24) |
| Wid/Sep/Div | 35 | 20 | 2.59[c] | (1.40–4.79) | 36 | 35 | 0.83 | (0.47–1.47) |
| Single | 37 | 34 | 1.61 | (0.88–2.94) | 36 | 37 | 0.95 | (0.52–1.71) |
| Age left full-time education | | | | | | | | |
| 13–15 | 44 | 52 | 1.00 | | 68 | 44 | 1.00 | |
| 16–18 | 104 | 121 | 0.88 | (0.52–1.52) | 110 | 104 | 0.66 | (0.39–1.12) |
| 19–21 | 19 | 18 | 1.10 | (0.50–2.43) | 13 | 19 | 0.40 | (0.17–0.93) |
| 22+ | 14 | 12 | 1.24 | (0.51–3.02) | 8 | 14 | 0.34[e] | (0.13–0.90) |
| Age at menarche | | | | | | | | |
| ≤11 | 24 | 45 | 1.00 | | 37 | 24 | 1.00 | |
| 12 | 27 | 28 | 1.68 | (0.81–3.51) | 42 | 27 | 1.03 | (0.51–2.09) |
| 13 | 57 | 56 | 1.85 | (1.00–3.45) | 50 | 57 | 0.55 | (0.27–1.04) |
| 14 | 45 | 43 | 1.90 | (0.99–3.62) | 39 | 45 | 0.53 | (0.27–1.04) |
| 15+ | 28 | 31 | 1.61 | (0.79–3.28) | 31 | 28 | 0.71 | (0.34–1.47) |
| Number of full-term pregnancies | | | | | | | | |
| 0 | 71 | 64 | 1.00 | | 63 | 71 | 1.00 | |
| 1 | 33 | 37 | 0.79 | (0.44–1.42) | 46 | 33 | 1.57 | (0.88–2.77) |
| 2 | 51 | 68 | 0.68 | (0.40–1.17) | 49 | 51 | 1.13 | (0.64–1.99) |
| 3+ | 26 | 34 | 0.69 | (0.35–1.38) | 41 | 26 | 1.90 | (0.94–3.85) |
| Age at first birth | | | | | | | | |
| 15–19 | 28 | 35 | 1.00 | | 41 | 28 | 1.00 | |
| 20+ | 82 | 104 | 0.93 | (0.51–1.71) | 95 | 82 | 0.74 | (0.41–1.33) |
| Ever had a spontaneous abortion | | | | | | | | |
| No | 152 | 185 | 1.00 | | 169 | 152 | 1.00 | |
| Yes | 29 | 18 | 2.24[b] | (1.17–4.30) | 30 | 29 | 0.95 | (0.54–1.68) |
| Ever had an induced abortion | | | | | | | | |
| No | 154 | 189 | 1.00 | | 176 | 154 | 1.00 | |
| Yes | 27 | 14 | 2.35[b] | (1.18–4.67) | 23 | 27 | 0.75 | (0.41–1.36) |
| Age at first intercourse | | | | | | | | |
| ≤16 | 52 | 58 | 1.00 | | 87 | 52 | 1.00 | |
| 17–20 | 104 | 113 | 1.08 | (0.67–1.74) | 98 | 104 | 0.51 | (0.32–0.80) |
| 21+ | 25 | 32 | 0.90 | (0.45–1.84) | 14 | 25 | 0.25[g] | (0.11–0.57) |
| Total number of sexual partners | | | | | | | | |
| 1 | 39 | 83 | 1.00 | | 39 | 39 | 1.00 | |
| 2–5 | 100 | 93 | 2.28 | (1.40–3.70) | 121 | 100 | 1.24 | (0.72–2.12) |
| 6+ | 40 | 25 | 3.52[f] | (1.84–6.76) | 39 | 40 | 0.96 | (0.50–1.84) |
| Number of regular partners | | | | | | | | |
| 1 | 47 | 93 | 1.00 | | 46 | 47 | 1.00 | |
| 2 | 50 | 57 | 1.79 | (1.06–3.04) | 82 | 50 | 1.66 | (0.96–2.88) |
| 3 | 49 | 26 | 3.82 | (2.07–7.05) | 39 | 49 | 0.82 | (0.45–1.51) |
| 4+ | 34 | 26 | 2.65[h] | (1.41–4.99) | 32 | 34 | 0.91 | (0.48–1.75) |
| Years since start of latest regular relationship | | | | | | | | |
| <2 | 50 | 21 | 1.00 | | 24 | 50 | 1.00 | |
| 2–5 | 56 | 37 | 0.72 | (0.37–1.39) | 48 | 56 | 1.65 | (0.87–3.13) |
| 6+ | 74 | 144 | 0.19[h] | (0.10–0.35) | 127 | 74 | 4.06[h] | (2.21–7.44) |
| Ever had a sexually transmitted disease | | | | | | | | |
| No | 100 | 108 | 1.00 | | 102 | 100 | 1.00 | |
| Yes | 81 | 95 | 0.91 | (0.61–1.37) | 97 | 81 | 1.16 | (0.77–1.74) |
| Ever had a partner with genital warts | | | | | | | | |
| No | 170 | 199 | 1.00 | | 187 | 170 | 1.00 | |
| Yes | 11 | 4 | 3.01 | (0.95–9.60) | 12 | 11 | 1.04 | (0.44–2.42) |
| Months using barrier contraception | | | | | | | | |
| 0 | 101 | 95 | 1.00 | | 110 | 101 | 1.00 | |
| 1–12 | 28 | 26 | 1.00 | (0.55–1.84) | 29 | 28 | 0.91 | (0.50–1.64) |
| 13–48 | 24 | 38 | 0.59 | (0.33–1.06) | 32 | 24 | 1.20 | (0.66–2.19) |
| 49+ | 28 | 44 | 0.64 | (0.36–1.14) | 28 | 28 | 0.93 | (0.51–1.70) |
| Oral contraceptive use | | | | | | | | |
| Never | 35 | 38 | 1.00 | | 32 | 35 | 1.00 | |
| Ex-user | 96 | 116 | 0.78 | (0.43–1.43) | 108 | 96 | 1.15 | (0.63–2.10) |
| Current user | 50 | 49 | 0.95 | (0.48–1.89) | 59 | 50 | 1.28 | (0.66–2.50) |
| Ever smoked | | | | | | | | |
| No | 92 | 98 | 1.00 | | 65 | 92 | 1.00 | |
| Yes | 89 | 105 | 0.90 | (0.60–1.35) | 134 | 89 | 2.20[d] | (1.44–3.35) |

[a]Stratified by 5-year age group; [b]$P < 0.05$; [c]$P < 0.005$; [d]$P < 0.0005$; [e]$P$ for trend < 0.01; [f]$P$ for trend < 0.001; [g]$P$ for trend < 0.0005; [h]$P$ for trend < 0.0001

*© 2000 Cancer Research Campaign*

were successfully interviewed. Among the interviewed cases there were 33 (14%) whose index spatula tested HPV-negative with the L1 consensus primer system in spite of multiple attempts at amplification under different conditions. These women did not differ from the HPV-positive cases with regard to age, cytology results, social class, sexual, obstetric or contraceptive history. They were 50% more likely than HPV-positive cases to have smoked but this difference was not significant ($\chi^2_{(1df)}$ = 0.63, $P$ = 0.43). PCR using different primers has not yet been attempted with these samples and it is not clear whether they represent truly HPV-unrelated CIN3 or if they are false-negatives. Because of this uncertainty these cases have been excluded from the current analyses. The data presented thus relate to 199 HPV-positive cases of CIN3, 181 HPV-positive controls and 203 HPV-negative controls.

The refusal rate was low and similar in cases and controls (15% and 13% respectively) and was unrelated to age. The main reason for failure to interview was inability to trace the woman (18% overall). Untraced women were those whose medical records and health authority registers recorded them as being still resident, but whom the interviewers found not to be at their registered address. Among both cases and controls untraced women were significantly younger than those interviewed, reflecting the high mobility of a fit young urban population; $\chi^2_{(1df)}$ for the difference between women under 30 and over 30 years was 8.67 ($P$ < 0.005) for the cases and 15.13 ($P$ < 0.0005) for controls. Significantly more controls (20%) than cases (11%) remained untraced at the end of the study ($\chi^2_{(1df)}$ for the difference = 12.81, $P$ < 0.0005), probably because the cases were under continuing medical surveillance and hence their records were more accurate. Four controls (2 HPV infected, 2 uninfected) who did not answer all the sexual questions and one HPV negative control who reported no regular partners were excluded from multivariate analyses involving these variables (see Table 5).

Table 1 shows the age distributions of cases and controls. These have been balanced by the stratified sampling process and reflect the age distribution of participating CIN3 cases. All the cases were diagnosed as the direct result of an abnormal smear, and as this study was conducted early in the history of the cohort it was the index smear that was abnormal in each case, 91 (46%) showing severe dyskaryosis and 108 (54%) showing lower grades of abnormality from atypia to moderate dyskaryosis. Among the HPV-positive controls 22 (12%) had an index smear showing a transient mild abnormality that resolved within a year. A further 22 had persistent dyskaryosis and of these 11 had mild or moderate dysplasia found at biopsy. Cytological abnormality was rare in uninfected women, seven (3.5%) having transient dyskaryosis and one having reportedly persistent severe dyskaryosis that showed CIN1 on biopsy.

Table 2 shows the distributions of cases and controls according to selected characteristics and risk factors. Odds ratios are stratified by age according to the 5-year strata used for sampling, but are otherwise unadjusted. In none of the analyses was there any evidence of heterogeneity of risk according to age. Among the controls risk of HPV infection was very strongly related to sexual behaviour. Both a woman's total lifetime number of sexual partners and her number of regular (longer than 3 months) relationships were highly predictive of risk ($P$ for trend < 0.001). HPV-infected women were also much more likely than uninfected controls to have commenced a new regular relationship within the past year. There was a moderately significant association between infection and history of miscarriage that was consistent in all analyses, remaining essentially unchanged after multivariate regression (see Table 5). No other risk factors for HPV infection were identified. The associations between marital status, therapeutic abortion and HPV infection disappeared after adjustment for number of partners and recency of the latest relationship. There was no association in this data set between cervical HPV infection and history of other sexually transmitted diseases (trichomonas, genital warts, genital herpes or gonorrhoea), use of barrier contraception, oral contraceptives (OCs) or smoking.

In the comparison of HPV-positive CIN3 cases against HPV-positive controls the only important determinants of disease were

**Table 3** Univariate odds ratios[a] for different smoking variables

| | HPV+ve Controls vs HPV–ve Controls | | | | HPV+ve Cases vs HPV+ve Controls | | | |
|---|---|---|---|---|---|---|---|---|
| | +ve | –ve | OR | 95% CI | Ca | Con | OR | 95%CI |
| Ever smoked | | | | | | | | |
| No | 92 | 98 | 1.00 | | 65 | 92 | 1.00 | |
| Yes | 89 | 105 | 0.90 | (0.60–1.35) | 134 | 89 | 2.20[b] | (1.44–3.35) |
| Current daily smoking habit (cigarettes/day) | | | | | | | | |
| Never | 92 | 98 | 1.00 | | 65 | 92 | 1.00 | |
| Ex | 15 | 27 | 0.66 | (0.32–1.35) | 22 | 15 | 2.14 | (1.03–4.45) |
| 1–19 | 35 | 45 | 0.79 | (0.46–1.34) | 38 | 35 | 1.58 | (0.88–2.84) |
| 20+ | 39 | 33 | 1.23 | (0.72–2.11) | 74 | 39 | 2.71[c] | (1.64–4.50) |
| Duration of smoking (years) | | | | | | | | |
| Never | 92 | 98 | 1.00 | | 65 | 92 | 1.00 | |
| 1–9 | 26 | 25 | 1.07 | (0.54–2.11) | 30 | 26 | 1.79 | (0.89–3.60) |
| 10–19 | 43 | 46 | 1.02 | (0.61–1.72) | 63 | 43 | 1.97 | (1.17–3.32) |
| 20+ | 20 | 34 | 0.63 | (0.32–1.23) | 41 | 20 | 3.13[d] | (1.57–6.23) |
| Average no. of cigarettes/day when smoking | | | | | | | | |
| Never | 92 | 98 | 1.00 | | 65 | 92 | 1.00 | |
| 1–10 | 33 | 38 | 0.89 | (0.51–1.56) | 29 | 33 | 1.36 | (0.73–2.51) |
| 11–16 | 28 | 38 | 0.77 | (0.44–1.36) | 45 | 28 | 2.20 | (1.24–3.89) |
| 17+ | 28 | 29 | 1.10 | (0.60–2.00) | 60 | 28 | 3.06[e] | (1.77–5.31) |

[a]Stratified by 5-year age group; [b]$P$ < 0.0005; [c]$P$ for trend < 0.0005 among current smokers; [d]$P$ for trend < 0.0005; [e]$P$ for trend < 0.0001

 *© 2000 Cancer Research Campaign*

**Table 4** Univariate odds ratios[a] for different oral contraceptive variables

| | HPV+ve Controls vs HPV–ve Controls | | | | HPV+ve Cases vs HPV+ve Controls | | | |
|---|---|---|---|---|---|---|---|---|
| | **+ve** | **–ve** | **OR** | **95% CI** | **Ca** | **Con** | **OR** | **95%CI** |
| Current oral contraceptive use | | | | | | | | |
| Never | 35 | 38 | 1.00 | | 32 | 35 | 1.00 | |
| Ex | 96 | 116 | 0.78 | (0.43–1.43) | 108 | 96 | 1.15 | (0.63–2.10) |
| Current | 50 | 49 | 0.95 | (0.48–1.89) | 59 | 50 | 1.28 | (0.66–2.50) |
| Total months of use | | | | | | | | |
| Never | 35 | 38 | 1.00 | | 32 | 35 | 1.00 | |
| 1–47 | 38 | 51 | 0.66 | (0.33–1.33) | 42 | 38 | 1.19 | (0.58–2.43) |
| 48–95 | 56 | 55 | 0.94 | (0.48–1.84) | 43 | 56 | 0.76 | (0.38–1.53) |
| 96+ | 52 | 59 | 0.87 | (0.45–1.67) | 82 | 52 | 1.52 | (0.80–2.88) |
| Months since first started use | | | | | | | | |
| Never | 35 | 38 | 1.00 | | 32 | 35 | 1.00 | |
| 1–95 | 47 | 40 | 1.14 | (0.48–2.75) | 33 | 47 | 0.52 | (0.21–1.28) |
| 96–191 | 56 | 79 | 0.60 | (0.30–1.21) | 76 | 56 | 1.23 | (0.61–2.48) |
| 192+ | 43 | 46 | 0.93 | (0.47–1.84) | 58 | 43 | 1.41 | (0.71–2.79) |
| Use of any progesterone-only pill[b] | | | | | | | | |
| Never | 35 | 38 | 1.00 | | 32 | 35 | 1.00 | |
| Ever | 17 | 9 | 1.67 | (0.62–4.48) | 17 | 17 | 1.29 | (0.48–3.44) |

[a]Stratified by 5-year age group; [b]Excludes women who have been on combined oral contraceptives, but never a progesterone-only pill

**Table 5** Adjusted odds ratios for HPV infection among controls, and CIN3 among those infected

| | HPV+ve Controls vs HPV–ve Controls | | | | HPV+ve Cases vs HPV+ve Controls | | | |
|---|---|---|---|---|---|---|---|---|
| | **+ve** | **–ve** | **OR** | **95% CI** | **Ca** | **Co** | **OR** | **95%CI** |
| Age at first intercourse | | | | | | | | |
| ≤16 | 52 | 57 | 1.00 | | 87 | 52 | 1.00 | |
| 17–20 | 102 | 112 | 1.08 | (0.64–1.83) | 98 | 102 | 0.57 | (0.35–0.94) |
| 21+ | 25 | 31 | 1.00 | (0.45–2.23) | 14 | 25 | 0.31[b] | (0.13–0.75) |
| Total number of sexual partners[a] | | | | | | | | |
| 1 | 39 | 83 | 1.00 | | 39 | 39 | 1.00 | |
| 2–5 | 100 | 93 | 2.15 | (1.30–3.54) | 121 | 100 | 0.88 | (0.50–1.57) |
| 6+ | 40 | 24 | 3.89[c] | (1.99–7.62) | 39 | 40 | 0.64 | (0.32–1.28) |
| Years since start of latest regular relationship | | | | | | | | |
| <2 | 50 | 21 | 1.00 | | 24 | 50 | 1.00 | |
| 2–5 | 56 | 36 | 0.77 | (0.39–1.53) | 48 | 56 | 1.72 | (0.88–3.35) |
| 6+ | 73 | 143 | 0.24[c] | (0.12–0.47) | 127 | 73 | 4.94[c] | (2.51–9.71) |
| Current daily smoking habit | | | | | | | | |
| Never | 91 | 96 | 1.00 | | 65 | 91 | 1.00 | |
| Ex | 14 | 26 | 0.69 | (0.31–1.53) | 22 | 14 | 1.69 | (0.76–3.75) |
| 1–19 | 35 | 45 | 0.79 | (0.44–1.41) | 38 | 35 | 1.48 | (0.79–2.76) |
| 20+ | 39 | 33 | 0.94 | (0.52–1.70) | 74 | 39 | 2.57[d] | (1.49–4.45) |
| Ever had a spontaneous abortion | | | | | | | | |
| No | 150 | 182 | 1.00 | | 169 | 150 | 1.00 | |
| Yes | 29 | 18 | 2.59[e] | (1.28–5.21) | 30 | 29 | 0.84 | (0.45–1.56) |

Odds ratios are stratified by age and also adjusted for the other variables in the table, *except:* [a] number of partners is not adjusted for years since start of latest regular relationship (see text). [b]$P$ for trend < 0.005; [c]$P$ for trend < 0.0005; [d]$P$ for trend (in current smokers) < 0.001; [e]$P$ < 0.01

age at first intercourse, long duration of the latest regular sexual relationship, and smoking. A moderate association with educational level and parity disappeared after adjustment for these variables. These findings are in complete contrast to the risk factors for infection per se. The association between disease and time since start of the most recent relationship, which is very strong and the inverse of that seen in the previous analysis, suggests that CIN3 is associated with longstanding persistent HPV infection.

Table 3 shows the results of further analyses of different measures of smoking habit. Among HPV-positive women there was strong evidence for a disease association with highly significant increasing trends in risk with increasing duration of smoking or number of cigarettes smoked per day. Modelling the variables simultaneously showed both dose measures to be important, although number of cigarettes per day was a stronger predictor of risk. In none of the analyses was an association found between risk of HPV infection and smoking.

Table 4 shows the results of further investigations of oral contraceptive use. Among the controls there was no indication of any association between OCs and HPV infection. Point estimates for the highest categories of each measure of use were slightly raised in CIN3 cases compared with HPV-positive controls, but none reached conventional levels of significance and there were no significant trends.

© 2000 Cancer Research Campaign 

**Table 6** Risk of CIN3[a] by HPV genotype among infected women

| | HPV+ve Cases vs HPV+ve Controls | | | |
|---|---|---|---|---|
| | Ca | Con | OR | 95% CI |
| HPV Type | | | | |
| 16 | 132 | 55 | 1.00 | |
| 16 Related[b] | 37 | 33 | 0.48 | (0.27–0.86) |
| 18 & Related[c] | 20 | 31 | 0.27 | (0.14–0.52) |
| Other[d] | 10 | 62 | 0.06 | (0.03–0.13) |

[a]Odds ratios stratified by 5-year age group; [b]31, 33, 35, 52, 58; [c]18, 39, 45, 59; [d]all others, including generic probe-positive but type unidentified. When multiple HPV types were detected women have been categorized by their highest risk virus according to the ranking in the table

Table 5 shows adjusted odds ratios for the acquisition of HPV infection and for the development of CIN3 among those infected, for all variables significantly associated with either outcome in a multivariate model. The estimates are mutually adjusted, except that number of partners is not adjusted for time since the beginning of the latest sexual relationship. This is because in this data set, given monogamy, close age stratification and age at first intercourse, the date of the start of the (single) relationship is closely predictable. The results are little different from those in Table 2 and still show a striking division between factors related to HPV infection and those predictive of CIN3 in infected women.

The risk of CIN3 by HPV genotype among infected women is shown in Table 6. HPV16, by far the most prevalent single virus type, has been analysed separately. Other viruses have been have been grouped according to their phylogeny based on nucleotide and amino acid sequence alignments of E6 sequences as published by the Human Papillomavirus Database at Los Alamos National Laboratory (http//hpv-web.lanl.gov) (van Ranst et al, 1993). The risk of CIN3 was far greater with HPV16 infection than with any other type, including related genotypes.

### DISCUSSION

Our most remarkable finding is the striking difference between the sexual risk factors that predict HPV infection and those that predict CIN3 among infected women. A woman's likelihood of having a detectable HPV infection depends only on her number of sexual partners and how recently she has acquired a new partner. The number of partners presumably determines the probability that she has ever been infected. Most relationships would have ended many years earlier, and the fact that they still predict her risk of a usually transient infection suggests that many such episodes are recurrences of latent long-standing infection. In contrast, HPV acquired from a new partner is likely to be an initial infection rather than a recurrence. The main risk factor for CIN3 among infected women is age at first intercourse, presumably because duration of exposure is the main risk factor for neoplastic progression. The fact that number of partners does not increase the CIN3 risk suggests that once a woman has been infected the risk is not increased by reinfection. The CIN3 risk in infected women who have not had a recent new partner is presumably high because diagnosis of CIN3 is often preceded by a period of persistent HPV detectability which may well be an effect of subclinical neoplastic progression rather than its cause. This risk factor raises a novel epidemiological difficulty. We observed a marked protective effect for HPV infection (OR 0.24) but an increased risk for CIN3 among those infected (OR 4.94) in women who had not acquired a new partner in the last 6 years. The corresponding OR for CIN3 using the HPV-negative controls would be the product of these estimates, which is close to unity. These highly significant opposite effects are informative in relation to the natural history of HPV infection and neoplasia, but they illustrate a potentially serious error in the analysis of such data. If we had chosen random controls irrespective of their HPV status, most of whom would be HPV-negative, an unadjusted analysis would have given the correct result that recent sexual behaviour has little effect on the CIN3 risk; but the same analysis stratified by HPV status would be dominated by the comparison of CIN3 cases against HPV-positive controls and would indicate that a woman's risk of developing CIN3 decreases abruptly when she begins a new relationship. This curious statistical artefact arises because most HPV infections are transient (Hinchliffe et al, 1995) but those preceding CIN3 diagnosis are persistent (Remmink et al, 1995). A transient infection acquired from a new partner is usually harmless, at least in the short term, but the minority of women in long-standing relationships who are still infected have persistent disease, and are thus at high risk of developing CIN3.

Over 99% of the women we interviewed were willing, in confidence, to discuss their sexual history. This, together with the very low and comparable refusal rate among both cases and controls leads us to anticipate minimal information bias in the study. A potential bias, however, is the differential loss of mobile first choice controls (movers): significantly more HPV-positive controls (27%) than HPV-negative controls (12%) or CIN3 cases (11%) had moved and were still untraced at the end of the study. It appears likely that women who move frequently and who do not re-register with a new doctor are less likely to be in a permanent or long-term relationship. We would anticipate that any bias caused by this selection, therefore, would tend to weaken all the duration effects we found and so our relative risk estimates are probably conservative.

Our analysis of CIN3 incidence in the whole Manchester cohort (Peto et al, in preparation) shows that CIN3 can develop quite rapidly in cytologically normal HPV carriers. Such women have a risk of CIN3 being detected at the following smear of between 5 and 10%. This observation, together with the findings here, indicates that the natural history of CIN3 often includes a long period of cytologically undetectable HPV infection.

The significant association between HPV infection and induced abortion shown in Table 2 disappeared after adjustment for the variables shown in Table 5, which reduced this OR from 2.35 ($P$<0.05) to 1.38 (not significant). Similar findings were previously reported by Karlsson et al. (1995). For spontaneous abortion, however, the same adjustment increased the OR from 2.24 ($P$<0.05) to 2.59 (P<0.01). This association between current HPV infection and previous spontaneous abortion supports the inference that many prevalent HPV infections are persistent or recurrent rather than newly acquired.

Our results show no clear association of oral contraceptive use with either HPV infection or disease. This is in contrast to a recent study (Ylitalo et al, 1999) where current use was associated with a highly significant four-fold increase in risk of CIN3. From Table 4 it can be seen that HPV-infected women who used the pill for 8 years or more, and those whose first use was more than 8 years earlier, were at marginally increased although not significant risk of CIN3. We have therefore analysed duration of use more than 8 years before diagnosis. The relative risk was 1.67 (1.03–2.72) in ever users, but there was no significant trend with duration of use.

 *© 2000 Cancer Research Campaign*

This risk was reduced to 1.47 (0.89–2.43) when duration of latest relationship, which is correlated with long-term pill use, was included in the model, and further reduced to 1.24 (0.72–2.15) when adjusted for the other variables listed in Table 5.

The evidence that smoking increases the risk of CIN3 and cervical cancer, which has been accumulating over the last 25 years, has recently been reviewed by Szarewski and Cuzick (1998). They concluded that the increased risk seen in most studies was reduced but not eliminated by adjustment for age at first intercourse and number of sexual partners, the residual risk remaining at or above two-fold in current smokers and showing a dose–response in many studies. The causal basis of the link, however, is still controversial because of the marked correlation between smoking and early sexual behaviour. In our study, for example, the proportion of controls whose age at first sexual intercourse was 16 or less was 38% among women who had ever smoked and 19% among never smokers. The prevalence of HPV infection was also increased among smokers in several studies, although the association was reduced by adjustment for sexual variables (Ley et al, 1991; Bauer et al, 1993; Olsen et al, 1998). Szarewski and Cuzick (1998) noted that adjustment for HPV infection further reduced the effect of smoking on cervical neoplasia in some studies, raising further doubts about the causal role of smoking (Morrison et al, 1991; Olsen et al, 1998). The independence of smoking and HPV infection in this study and the consistent and highly significant dose-response for CIN3 compared with HPV-infected controls provide strong evidence that smoking acts synergistically with HPV to cause cervical neoplasia.

### ACKNOWLEDGEMENTS

Our thanks are due to all the clinic and laboratory staff who helped to construct the Manchester cohort, and to Heidi Bauer, Catherine Greer, Patti Gravitt and Cosette Wheeler for the provision of reagents and probes, and for their help in setting up our HPV laboratory. Most of all we thank the women, cases and controls, who so willingly agreed to participate and give interviews. Janet Wilson, together with Wendy Binns, performed the interviews, Michelle Haywood and N Elanko ran the HPV lab, and Jane Hatch and Doreen Lewis managed the data.

The study was supported and funded by the Cancer Research Campaign through project grants SP2285/0401 and SP2285/0501.

### REFERENCES

Bauer HM, Ting Y, Greer CE, Chambers JC, Tashiro CJ, Chimera J, Reingold A and Manos MM (1991) Genital human papillomavirus infection in female university students as determined by a PCR-based method. *JAMA* **265**: 472–477

Bauer HM, Greer CE and Manos MM (1992) Determination of genital human papillomavirus infection by consensus PCR amplification. In *Diagnostic molecular pathology: a practical approach*. Herrington CS and McGee JOD (eds), pp 131–152. Oxford University Press: Oxford

Bauer HM, Hildesheim A, Schiffman MH, Glass AG, Rush BB, Scott DR, Cadell DM, Kurman RJ and Manos MM (1993) Determinants of genital human papillomavirus infection in low-risk women in Portland, Oregon. *Sex Transm Dis* **20**: 274–278

Bosch FX, Manos MM, Munoz N, Sherman M, Jansen AM, Peto J, Schiffman MH, Moreno V, Kurman R and Shah KV. International Biological Study on Cervical Cancer (IBSCC) Study Group (1995) Prevalence of human papillomavirus in cervical cancer: a worldwide perspective. *J Natl Cancer Inst* **87**: 796–802

Breslow NE and Day NE (1980) *Statistical Methods in Cancer Research. Vol 1 – The analysis of case-control studies*. IARC Scientific Publications: Lyon

Brinton LA (1992) Epidemiology of cervical cancer – overview. In *The epidemiology of human papillomavirus and cervical cancer*. Munoz N, Bosch FX, Shah KV and Meheus A (eds), pp 3–23. International Agency for Research on Cancer: Lyon

Evander M, Edlund K, Gustafsson A, Jonsson M, Karlsson R, Rylander E and Wadell G (1995) Human papillomavirus infection is transient in young women: a population-based cohort study. *J Infect Dis* **171**: 1026–1030

Hildesheim A, Gravitt P, Schiffman MH, Kurman RJ, Barnes W, Jones S, Tchabo J-G, Brinton LA, Copeland C, Epp J and Manos MM (1993) Determinants of genital human papillomavirus infection in low-income women in Washington DC. *Sex Transm Dis* **20**: 279–285

Hinchliffe SA, van Velzen D, Korporaal H, Kok PL and Boon ME (1995) Transience of cervical HPV infection in sexually active young women with normal cervicovaginal cytology. *Br J Cancer* **72**: 943–945

Ho GYF, Bierman R, Beardsley L, Chang CJ and Burk RD (1998) Natural history of cervicovaginal papillomavirus infection in young women. *N Engl J Med* **338**: 423–428

IARC (1995) *Monographs on the evaluation of carcinogenic risks to humans. Vol 64 Human papillomaviruses*. International Agency for Research on Cancer: Lyon

Karlsson R, Jonsson M, Edlund K, Evander M, Gustavsson A, Boden E, Rylander E and Wadell G (1995) Lifetime number of partners as the only independent risk factor for human papillomavirus infection: a population-based study. *Sex Transm Dis* **22**: 119–127

Layde PM (1989) Smoking and cervical cancer: cause or coincidence? *JAMA* **261**: 1631–1633

Ley C, Bauer HM, Reingold A, Schiffman MH, Chambers JC, Tashiro CJ and Manos MM (1991) Determinants of genital human papillomavirus infection in young women. *J Natl Cancer Inst* **83**: 997–1003

Manos MM, Ting Y, Wright DK, Lewis AJ, Broker TR and Wolinsky SM (1989) The use of polymerase chain reaction amplification for the detection of genital human papillomaviruses. *Molecular Diagnostics of Human Cancer: Cancer Cells* **7**: 209–214

Melkert PWJ, Hopman E, van den Brule AJC, Risse EKJ, van Diest PJ, Bleker OP, Helmerhorst T, Schipper MEI, Meijer CJLM and Walboomers JMM (1993) Prevalence of HPV in cytomorphologically normal cervical smears, as determined by the polymerase chain reaction, is age-dependent. *Int J Cancer* **53**: 919–923

Morrison EAB, Ho GYF, Vermund SH, Goldberg GL, Kadish AS, Kelley KF and Burk RD (1991) Human papillomavirus infection and other risk factors for cervical neoplasia: a case-contol study. *Int J Cancer* **49**: 6–13

Olsen AO, Dillner J, Skrondal A and Magnus P (1998) Combined effect of smoking and human papillomavirus type 16 infection in cervical carcinogenesis. *Epidemiology* **9**: 346–349

Phillips AN and Davey Smith G (1994) Cigarette smoking as a potential cause of cervical cancer: has confounding been controlled? *Int J Epidemiol* **23**: 42–49

Remmink AJ, Walboomers JMM, Helmerhorst TJM, Voorhorst FJ, Rozendaal L, Risse EKJ, Meijer CJLM and Kenemans P (1995) The presence of persistent high-risk HPV genotypes in dysplastic cervical lesions is associated with progressive disease: natural history up to 36 months. *Int J Cancer* **61**: 306–311

Schiffman MH (1992) Recent progress in defining the epidemiology of human papillomavirus infection and cervical neoplasia. *J Natl Cancer Inst* **84**: 394–398

Szarewski A and Cuzick J (1998) Smoking and cervical neoplasia: a review of the evidence. *J Epidemiol Biostat* **3**: 229–256

van Ranst MA, Tachezy R, Delius H and Burk RD (1993) Taxonomy of the human papillomaviruses. *Papillomavirus Reports* **4**: 61–65

Walboomers JMM, Jacobs MV, Manos MM, Bosch FX, Kummer JA, Shah KV, Snijders PJF, Peto J, Meijer CJLM and Munoz N (1999) Human papillomavirus is a necessary cause of invasive cervical cancer worldwide. *J Pathol* **189**: 12–19

Wheeler CM, Parmenter CA, Hunt WC, Becker TM, Greer CE, Hildesheim A and Manos MM (1993) Determinants of genital human papillomavirus infection among cytologically normal women attending the University of New Mexico Student Health Center. *Sex Transm Dis* **20**: 286–289

Ylitalo N, Sorensen P, Josefsson A, Frisch M, Sparen P, Ponten J, Gyllensten U, Melbye M and Adami H-O (1999) Smoking and oral contraceptives as risk factors for cervical carcinoma in situ. *Int J Cancer* **81**: 357–365

*© 2000 Cancer Research Campaign* 

### APPENDIX

### The Manchester UK cohort: clinical collaborators

**GP Practices:**
Drs D Bodey, S Hollingshead and T Rashid, Wilmslow Road, Fallowfield;
Drs H Burgess, S Goodman and HC Sutherland, Mauldeth Road, Fallowfield;
Drs K Checkland, M Jones and K Wells, Church Street, Marple, Stockport;
Dr DN Riley, Robins Lane Medical Centre, Bramhall;
Drs E Tierney, JCV Gillighan, JF Downie and AP Morris, Whitworth Medical Centre, Rochdale;
Partners of the Northenden Group Practice, Palatine Road, Withington;
Partners of the Robert Darbyshire Practice, Rusholme Health Centre
**Family planning clinics**:
Adswood Clinic, Stockport; Ancoats Hospital; Brinnington Clinic, Stockport; Cheadle Health Clinic, Stockport; Corporation Road Clinic, Eccles; Grasmere Street Health Centre, Leigh; Hazel Grove Clinic, Stockport; Heaton Norris Clinic, Stockport; Higher Broughton Health Centre, Salford; Hope Hospital, Salford; Lanceburn Health Centre, Salford; Langworthy Clinic, Salford; Little Hulton Clinic, Worsley; Liverpool Road Clinic, Hindley; Longsight Health Centre; Lower Broughton Clinic, Salford; Maple Clinic, Stockport; North Reddish Clinic, Stockport; Northern Hospital, Cheetham; Offerton Clinic Stockport; Offerton Green Clinic, Stockport; Partington Lane Clinic, Swinton; Romiley Health Centre, Stockport; Shaw Health Clinic, Stockport; St Mary's Hospital; Stepping Hill Clinic, Stockport; The Palatine Centre, Palatine Road, Withington; Woodley Clinic, Stockport
**Screening clinics**:
Abbey Hey Clinic, Gorton; Alexandra Park Health Centre; Ancoats Community Clinic; Assheton Road Clinic, Newton Heath; Atherton Clinic, Wigan; Baguley Clinic; Beech Hill Health Centre, Wigan; Beswick Health Centre; Bevendon Square Clinic, Salford; Boothtown Clinic; Bramhall Clinic, Stockport; Bredbury Clinic, Stockport; Brookfield Clinics, Stockport; Brunswick Clinic; Burley House Clinic, Stockport; Central Drive Clinic, Crumpsall; Charlestown Health Centre, Blackley; Child Health Centre, Benchill; Clayton Health Centre; College Road Clinic, Leigh; Corporation Road Clinic, Eccles; Crumpsall Clinic; Derby Road Clinic, Golborne; Derbyshire House Clinic; Gorton Combined Clinic, West Gorton; Grasmere Street Health Centre, Leigh; Haig Road Clinic, Aspull; Harpurhey Health Centre; Hazel Grove Clinic, Stockport; Heaton Norris Clinic, Stockport; Hulme Clinic; Irlam Health Centre; Levenshulme Health Centre; Little Hulton Clinic, Worsley; Liverpool Road Health Centre, Hindley; Liverpool Street Clinic, Salford; Longshoot Lane Health Centre, Scholes; Lower Broughton Clinic, Salford; Monton Road Clinic, Eccles; Moss Side Health Centre; Newton Heath Health Centre; Nicholas Road Health Centre, Chorlton; North Reddish Clinic, Stockport; Offerton Clinic, Stockport; Ordsall Health Centre, Salford; Orrell Road Clinic, Wigan; Partington Lane Clinic, Swinton; Pemberton Clinic, Wigan; Plant Hill Clinic, Blackley; Platt Bridge Clinic, Wigan; Poplar Street Clinic, Tyldesley; Queens Road Clinic, Ashton-in-Makerfield; Romiley Clinic, Stockport; Smedley Street Clinic, Crumpsall; Standish Clinic, Wigan; Varley Street Clinic, Miles Platting; Walkden Clinic; Walmer Street Clinic, Rusholme; Wellwoman Clinic, Woodhouse Park; Wellwoman Clinic, Irlam; Wellwoman Clinic, Stockport; Wilmslow Road Clinic, Didsbury; Woodhouse Park Clinic, Wythenshawe

*© 2000 Cancer Research Campaign*
